# First fully endoscopic metabolic procedure with NOTES gastrojejunostomy, controlled bypass length and duodenal exclusion: a 9-month porcine study

**DOI:** 10.1038/s41598-021-02921-9

**Published:** 2022-01-07

**Authors:** Jean-Michel Gonzalez, Sohaib Ouazzani, Laurent Monino, Laura Beyer-Berjot, Stephane Berdah, Nicolas Cauche, Cecilia Delattre, Joyce A. Peetermans, Peter Dayton, Ornela Gjata, Darren Curran, Marc Barthet

**Affiliations:** 1 Department of Hepatogastroenterology, Hôpital Nord, Assistance Publique-Hôpitaux de Marseille: Faculty of Medicine, Aix-Marseille University, Chemin des Bourrely, 13915 Marseille Cedex 20, France; 2 Department of Digestive Surgery, Hôpital Nord, Assistance Publique-Hôpitaux de Marseille, Aix-Marseille University, Marseille, France; 3 Centre for Surgical Teaching and Research (CERC), Aix-Marseille University, Marseille, France; 4 Brussels Medical Device Center (BMDC), Brussels, Belgium; 5 Endoscopy Division, Boston Scientific Corporation, Marlborough, MA USA

**Keywords:** Gastroenterology, Medical research

## Abstract

We conducted a pilot study of a potential endoscopic alternative to bariatric surgery. We developed a Natural Orifice Transluminal Endoscopic Surgery (NOTES) gastric bypass with controlled bypass limb length using four new devices including a dedicated lumen-apposing metal stent (GJ-LAMS) and pyloric duodenal exclusion device (DED). We evaluated procedural technical success, weight change from baseline, and adverse events in growing Landrace/Large-White pigs through 38 weeks after GJ-LAMS placement. Six pigs (age 2.5 months, mean baseline weight 26.1 ± 2.7 kg) had initial GJ-LAMS placement with controlled bypass limb length, followed by DED placement at 2 weeks. Technical success was 100%. GJ-LAMS migrated in 3 of 6, and DED migrated in 3 of 5 surviving pigs after mucosal abrasion. One pig died by Day 94. At 38 weeks, necropsy showed 100–240 cm limb length except for one at 760 cm. Weight gain was significantly lower in the pigs that underwent endoscopic bypass procedures compared to expected weight for age. This first survival study of a fully endoscopic controlled bypass length gastrojejunostomy with duodenal exclusion in a growing porcine model showed high technical success but significant adverse events. Future studies will include procedural and device optimizations and comparison to a control group.

## Introduction

In addition to lifestyle modifications, bariatric surgery (Roux-en-Y gastric bypass (RYGB), sleeve gastrectomy, biliopancreatic diversion or laparoscopic adjustable gastric band) is recommended for adult patients with BMI ≥ 40 or with BMI ≥ 35 with one or more severe obesity-related comorbidities, including type 2 diabetes mellitus or non-alcoholic fatty liver disease^1^. Bariatric surgery can reduce type 2 diabetes mellitus^2,3^, non-alcoholic fatty liver disease^4^, and hypertension in obese patients^5^ but may have a 10–17% risk of postoperative complications^6,7^. Because less than 1% of patients who qualify for bariatric surgery based on the above-mentioned criteria are estimated to undergo bariatric surgery worldwide^8^, there is a need for safe and effective alternatives to surgery.

Since the 1980s when endoscopically placed intragastric balloons were introduced, endoscopic alternatives to bariatric surgery have been investigated to promote weight loss and improve metabolic parameters in obese patients who are ineligible for bariatric surgery, or as a bridge to surgery for patients who are anticipated to become eligible after presurgical weight loss^9^. Of interest recently, an endoscopic bariatric procedure via a natural orifice transluminal endoscopic surgery (NOTES) approach with self-expandable lumen-apposing metal stents (LAMS) has been explored with the goal of using a nonsurgical procedure to create a gastrojejunal anastomosis (GJA) to mimic the malabsorptive effect of an RYGB, first in a porcine model^10^ and then in a human feasibility study^11^. Use of a fully-covered stent for GJA by NOTES has been demonstrated to avoid GJA leakage^10,11^. A 2021 cross-sectional study of 3 pigs used a NOTES one anastomosis gastric bypass procedure^12^; however, longitudinal follow-up was not included to prospectively assess device reliability and durability, efficacy and safety^12^. Remaining challenges include optimizing the technique and associated devices for reliable creation of an endoscopic bypass. This includes reliable selection of the jejunal limb of a well-controlled length to maximize weight loss and metabolic effect, and creation of a reliable and durable GJA and duodenal exclusion.

The goal of the current exploratory pilot study was to demonstrate in a survival growing porcine model technical feasibility and safety of the fully endoscopic bypass procedure including 4 dedicated novel devices allowing NOTES GJA, measurement of the bypassed limb, duodenal obstruction. Weight and safety outcomes of the study animals were followed for 38 weeks, and procedural improvements were implemented during serial endoscopic examinations. Removability of the implanted devices was evaluated.

## Methods

### Procedural and device development and testing (Video 1)

The steps of the NOTES gastric bypass with controlled limb length were as follows.

1. Endoscopic measurement of the bypassed limb by jejunal insertion of a dedicated light beacon (150 cm target length)
2. Endoscopic creation of NOTES gastrojejunal anastomosis:
  Endoscopic gastrostomy.
  Finding into the peritoneal cavity of the light generated by the enteral light beacon through the jejunal wall.
  Grasping the targeted jejunum with a dedicated atraumatic grasper (Video 2).
  Insertion of a 20mm dedicated lumen-apposing metal stent (LAMS) (Video 3).
  Creating the GJA by pulling back the targeted jejunum toward the gastrostomy.
3. Occluding the pylorus with a dedicated device (DED) (Video 4).

**Figure 1:**
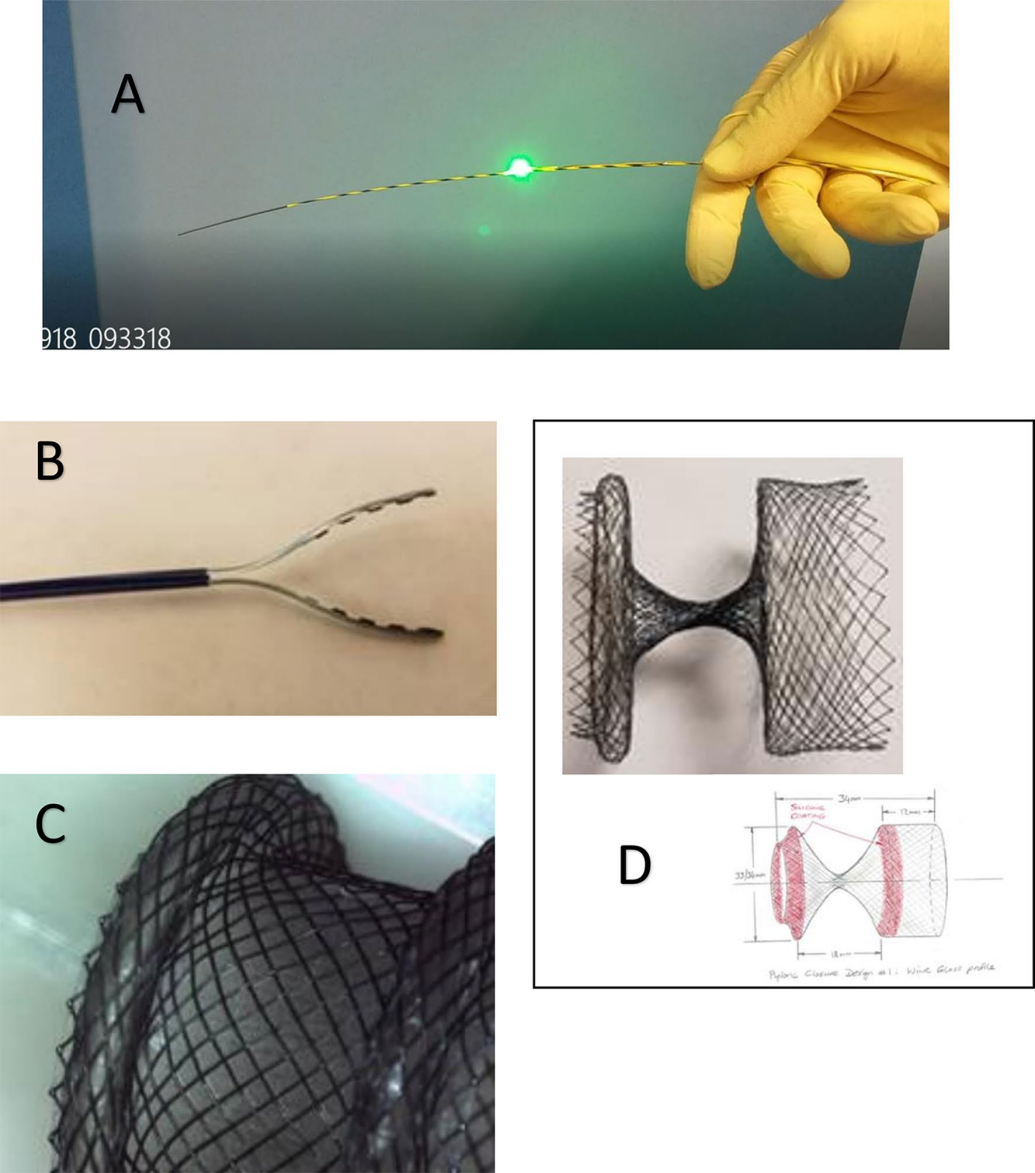
Final devices used in the fully endoscopic jejunal bypass with duodenal exclusion procedure. (**A**) Enteral Beacon. (**B**) Atraumatic Grasper. (**C**) GJ-LAMS. (**D**) DED (final size: 34 mm long with 34-mm diameter flange).

Device development and testing was accomplished in animal laboratories between April 2017 and September 2019 (“Supplementary Appendix”). Four individual devices were developed for the fully endoscopic jejunal bypass with duodenal exclusion: Enteral light Beacon, Atraumatic Grasper, Modified LAMS (GJ-LAMS), and Duodenal Exclusion Device (DED) (Boston Scientific Corporation, Marlborough, Massachusetts, USA and Brussels Medical Device Center, Brussels, Belgium) (Fig. 1A–D).

### Prospective fully endoscopic procedure and survival animal study

**Figure 2:**
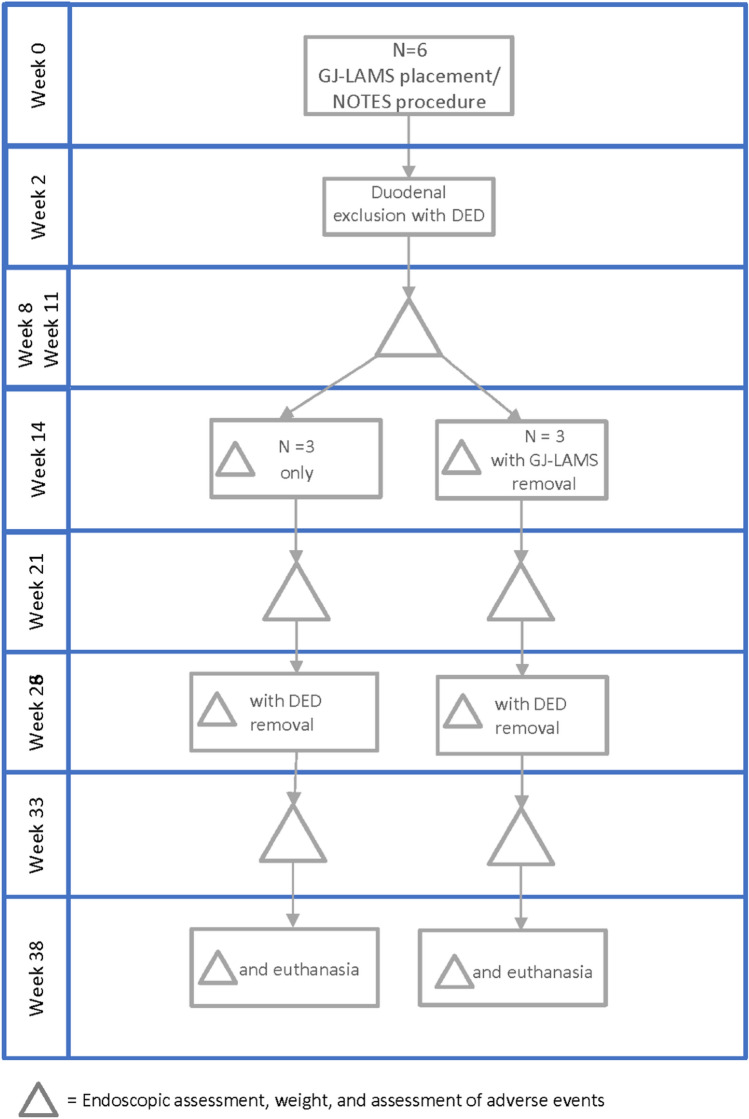
Planned GJ-LAMS and DED placement and removal. *GJ-LAMS* gastrojejunal lumen-apposing metal stent, *NOTES* natural orifice transluminal endoscopic surgery, *DED* duodenal exclusion device.

A 38-week prospective animal study was conducted between October 2019 and July 2020 by physician procedural experts at an academic animal research facility (Centre for Surgical Teaching and Research (CERC), Aix-Marseille University, Marseille, France) (Fig. 2). The French Ministry of Higher Education, Research and Innovation approved the study. All applicable institutional and/or national guidelines for the care and use of animals were followed under approval of the Institutional Animal Care and Use Committee (IACUC) and the ethical principles of the Canadian Council of Animal Care (CCAC). Efficacy (animal wellbeing, oral intake, weight) and safety outcomes are presented from baseline (GJ-LAMS placement) to 38 weeks. The study animals were 2.5-month-old healthy, normal-weight Landrace/Large-White domestic pigs acquired from a regional breeder (Gerald Moretti, St-Cannat, France). The pigs arrived at the animal facility 3 days before the procedures. Animal handling, preoperative care and anesthesia are documented in the “Supplementary Appendix”. The study was reported in accordance with the ARRIVE guidelines^13^.

### Initial placement of GJ-LAMS and DED

The fully endoscopic procedure involving placement of a GJ-LAMS and DED using a NOTES approach is graphically illustrated in Video 1. New techniques were developed to allow controlled length of the jejunal bypass using a light beacon as a target (Day 0), and pyloric closure using an endoscopically placed DED (Weeks 1–2). Placement times for the GJ-LAMS and (separately) DED were measured from endoscope insertion into the pig’s mouth to endoscope removal from the pig’s mouth after completion of the procedure. GJ-LAMS removal was planned for half of the pigs on Week 14. Removal of all DEDs was planned for all pigs at Week 28. In case of migration before the planned date of removal, the GJ-LAMS would be replaced if the GJA appeared narrowed upon endoscopic examination, and the DED would be replaced in all cases. After migration into the stomach of all DEDs originally placed, the protocol was modified at Week 11 to include mucosal abrasion immediately prior to deployment of a replacement DED.

### Monitoring and follow-up

Oral intake, animal wellbeing and symptoms were monitored daily per animal facility standard procedures. Pigs weights were recorded at each endoscopic intervention while the animals were anesthetized for procedures, namely on Weeks 0, 1–2, 8, 11, 14, 21, 28, 33 and 38 weeks. These timepoints coincide with endoscopic intervention either to place or remove GJ-LAMS or DED, or merely to assess GJ-LAMS and DED patency or possible migration.

### Final endoscopic examination

At 38 weeks, a final endoscopic examination was conducted on each pig, specifically observing for gastric stasis; inflammation, ulceration and patency at the GJA and DED sites; pylorus pattern; and duodenal inflammation, stenosis or patency.

### Euthanasia and necropsy examination

Euthanasia was performed after 38 weeks of follow-up after GJ-LAMS placement. In case of death before this time, postmortem endoscopic examination was performed to evaluate whether devices were in place and assess the appearance of adjacent tissue.

At necropsy, gross examination was performed of the peritoneal cavity to look for signs of inflammation, infection or adhesions; of the stomach and small bowel to look for inflammation or distention; and of the GJA and pylorus to look for inflammation, induration or adhesions. The bypass limb length was measured and jejunal tissue was examined for distension or strictures.

### Endpoints

Endpoints for the 38-week prospective study were (1) technical success (including placement and removal of GJ-LAMS and DED), (2) animal weight change from baseline, (3) device-related adverse events (AEs), and (4) gross examination findings at necropsy.

### Statistical analysis

Descriptive statistics included the mean, standard deviation (SD) and range for continuous variables (age, baseline weight and weight change from baseline) and the proportion (numerator over denominator) for binary variables such as AEs. Serial weights were plotted for each animal at the time of each endoscopic examination, from baseline to 38 weeks; follow-up weights were compared to baseline weight using a paired t-test. Observed weights were plotted versus corresponding weights from similar animals using the Gompertz model ± 12% predicted weight from Aubry et al.^14^, the weights reported in Tomović et al.^15^, and reference weights provided by Author MB (personal communication with breeder Gerald Moretti, St-Cannat, France). Statistical analyses were performed using SAS 9.4 software (SAS Institute Inc., Cary, NC, USA).

## Results

### Technical success

**Figure 3:**
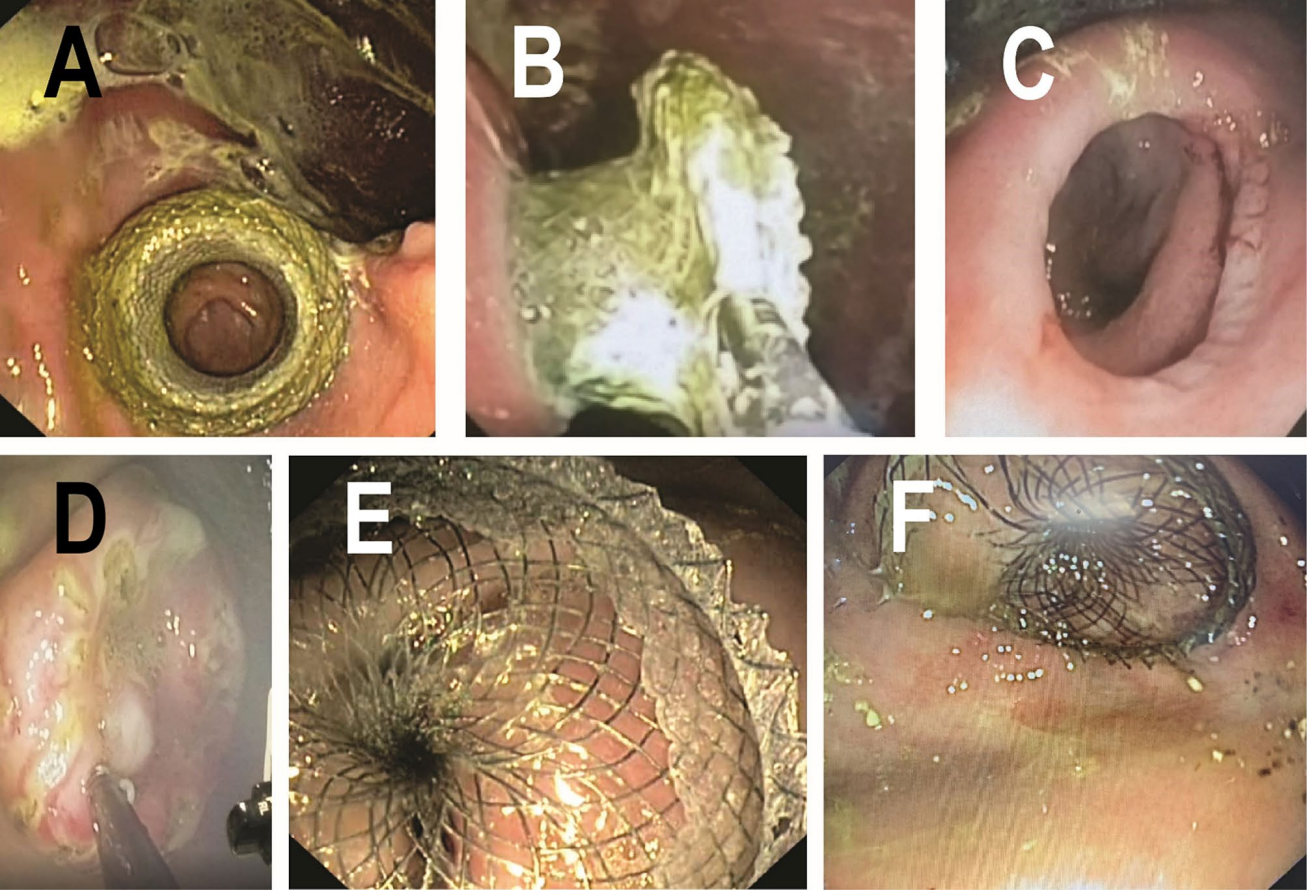
Steps in the fully endoscopic jejunal bypass with duodenal exclusion procedure. (**A**) View of jejunal wall though the recently deployed GJ-LAMS. (**B**) Atraumatic removal of GJ-LAMS with grasper after 14 weeks of GJ-LAMS indwell. (**C**) View of jejunal wall through mature GJA after removal of the GJ-LAMS at the end of 13 weeks of indwell. (**D**) Mucosal abrasion before DED placement at Week 11. (**E**) DED immediately after deployment in the pylorus at Week 1. (**F**) DED shortly before atraumatic removal after 22 weeks of indwell. Tissue ingrowth is evident.

**Figure 4:**
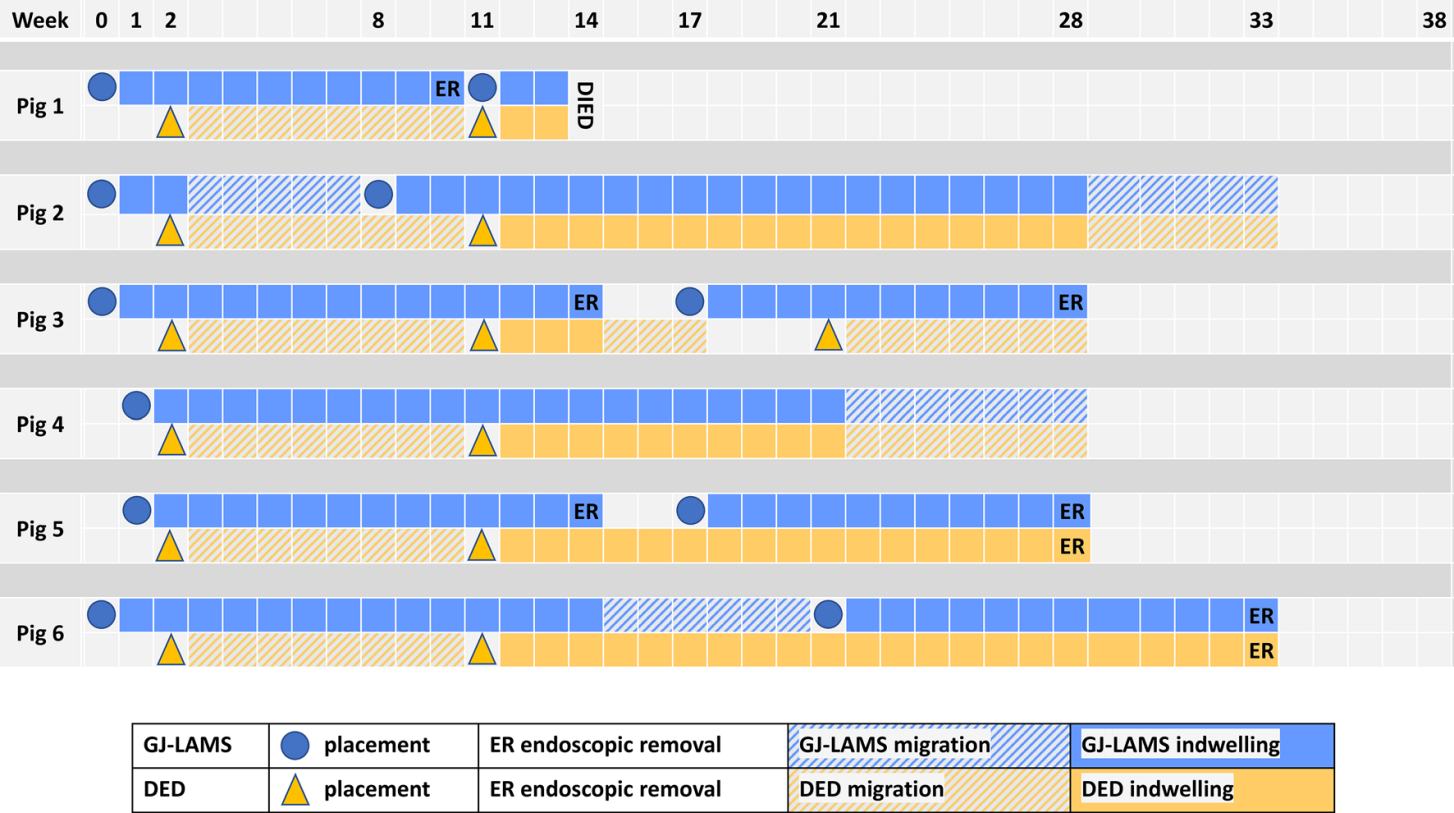
Placement, removal and migration of GJ-LAMS and DED in domestic pigs.

All (100%) GJAs and DEDs were completed on the first placement attempt, at Week 0 and Week 2 respectively (Table 1, Fig. 3A,D,E). Figure 4 graphically presents GJ-LAMS and DED placements and removals for each pig. In the five pigs that completed 38 weeks of follow-up, estimated cumulative GJ-LAMS indwell ranged from 91 to 231 days, and estimated cumulative DED indwell after placement with mucosal abrasion ranged from 14 to 154 days. In cases of migration, the estimated range of indwell duration assumed migration on the day of device placement for the minimum, and migration on the day the device was observed to no longer be indwelling for the maximum indwell time. Additional details are presented in the “Supplementary Appendix”.

**Table 1:** Animal and procedural measurements, endoscopic and necropsy findings.

Measurement	Mean ± SD or percent (n/N)
**Procedural characteristics**	
Placement time (min)^a^	
GJ-LAMS (including beacon time and stent placement time)	35.5 ± 9.2
DED (Week 2)	4.5 ± 1.9
DED (Week 11)	5.5 ± 1.2
Technical success^a^	
GJA using GJ-LAMS (Week 0–1)	100%
Duodenal exclusion by pyloric closure using DED (Weeks 2 and 11)	100%
**Findings between 0 and 38 weeks**	
Baseline age (months)	2.5
Weight (kg)	
Initial for 7 pigs	26.1 ± 2.7
Initial for 5 pigs that survived to Week 38^a^	26.5 ± 1.6
Final (38 weeks past GJ, 13 weeks past most recent pyloric closure)^a^	45.6 ± 12.3 (range 32.5, 60.8)
Weight change from baseline to 38 weeks^a^	19.1 ± 11.3 (range 6.3, 31.7)
Device-related adverse events^a^	
Total number of GJ-LAMS migrations during study	4
Mean number of GJ-LAMS migrations per pig	0.7 ± 0.8
Total number of DED migrations	10
Mean number of DED migrations	
Overall	1.7 ± 0.8 (6)
After mucosal abrasion was initiated^a^	0.8 ± 0.8 (5)
Other adverse events	
Transient diarrhea	42.9% (3/7)
Asymptomatic partial duodenal stenosis	28.6% (2/7)
Small wall abscesses in the muscularis externa^a^	40% (2/5)
Death (without evidence of device failure on necropsy)	28.6% (2/7)
**Endoscopic findings at 38 weeks**^a^	
Mucosa appears normal	
Esophagus	100.0% (5/5)
Gastric	100.0% (5/5)
Gastric stasis	
None	20% (1/5)
Slight	40% (2/5)
Much	40% (2/5)
GJA	
Mean diameter of GJA stoma (mm)	14.2 ± 3.9 (range 10.0, 20.0)
Pylorus	
Mean diameter of pyloric orifice (mm)	7.8 ± 4.5 (range 4.0, 14.0)

*GJ* gastrojejunal, *GJA* gastrojejunal anastomosis, *DED* duodenal exclusion device.

^a^Not including Pig 1 or original pig 4 (died). Baseline for pigs 1–3 and 6 was Day 0; baseline for pigs 4 and 5 was Day 7/Week 1.

### Pig follow-up and outcomes

During the follow-up, a total of 4 GJ-LAMS migrations occurred in 3 of the 6 animals during the follow-up period (Table 1, Fig. 4), and were replaced at the discretion of the endoscopist based on the patency of the GJA. The migrated GJ-LAMS passed uneventfully. Six (6) GJ-LAMS in 4 pigs were removed endoscopically uneventfully after 10, 11, 11, 12, 13, and 14 weeks of indwell (Fig. 3B).

A total of 10 DED migrations occurred among all 6 animals, i.e. 3 animals had 1, 2 animals had 2, and 1 animal had 3 DED migrations (Table 1, Fig. 4). DED migration occurred at 8 weeks follow-up in all the cases, all being without previous mucosal abrasion. Mucosal abrasion was initiated at the time of DED replacement (11 weeks and after), 1 pig died 3 weeks later, 3 pigs had 1 or 2 subsequent DED migrations, and 2 pigs had stable DED indwell until uneventful endoscopic removal after 17 and 22 weeks of indwell (Fig. 3F).

Narrowing of the GJA following GJ-LAMS migration or endoscopic removal was observed endoscopically in some instances (two cases after planned stent removal and three cases of spontaneous migration); these represented partial stenoses only. They were all treated by dilatation in three cases and stent reinsertion in two cases. Even at times when a DED was unambiguously in place, the pigs maintained standardized food intake. Oral nutrition was maintained in all pigs at all times except during preprocedure fasting and in the immediate postanesthesia recovery period.

During planned follow-up after GJA stent removal at Week 14, excellent patency was observed of the GJ-LAMS in pig #3 and pig #5. At Week 17, GJA stenosis was observed in pig #3 and pig #5; therefore, a new GJ-LAMS was placed through the remaining pinhole GJA in one pig. In the other pig a puncture was made through the fibrotic scar allowing insertion of a guidewire over which a new GJ-LAMS was placed. Final endoscopic removal of the GJ-LAMS was performed at Week 28 in pig #3 and pig #5 and at Week 33 in pig #6.

### Endoscopic evaluations at end of study

At the 38-week final endoscopic examination immediately prior to euthanasia and necropsy, all five surviving pigs had normal-appearing esophageal and gastric mucosa (Table 1). One pig (pig #2) had a narrowed GJA which was easily dilated endoscopically to 12 mm and one (pig #6) had a narrowed GJA of 10 mm diameter. The other three pigs had a patent GJA measured to be between 13 and 20 mm in diameter. Endoscopic estimates of the diameter of the pylorus ranged from 4 to 14 mm. In one pig (pig #4), the gastric side of the pylorus had signs of mucosal trauma and granuloma formation. There was gastric stasis of solid food in another pig (pig #5).

Mean diameter of the GJA stoma was 14.2 ± 3.9 mm and visualization of the jejunal wall was reported in four pigs (Fig. 3C). Mean diameter of the pyloric orifice was 7.8 ± 4.5 mm.

### Necropsy findings at end of study

Upon gross examination at necropsy, peritoneal adhesions were noted in 2 (40%) pigs, but no signs of inflammation, infection or leakage of gastric contents into the peritoneal cavity were seen (Table 1). Mean diameter of the GJA stoma was 13.2 ± 2.2 mm; all stomata appeared mature, and 1 (20.0%) (pig #2) appeared fibrotic. Mean diameter of the pyloric orifice was 8.2 ± 3.3 mm, and mean length of the pylorus was 0.6 ± 0.2 cm. One pig (pig #5) showed signs of duodenal scarring at the level of the second portion of the duodenum. No signs of small bowel stasis or distension were reported. Histopathological findings in tissue samples obtained at necropsy are described in the “Supplementary Appendix”.

**Figure 5:**
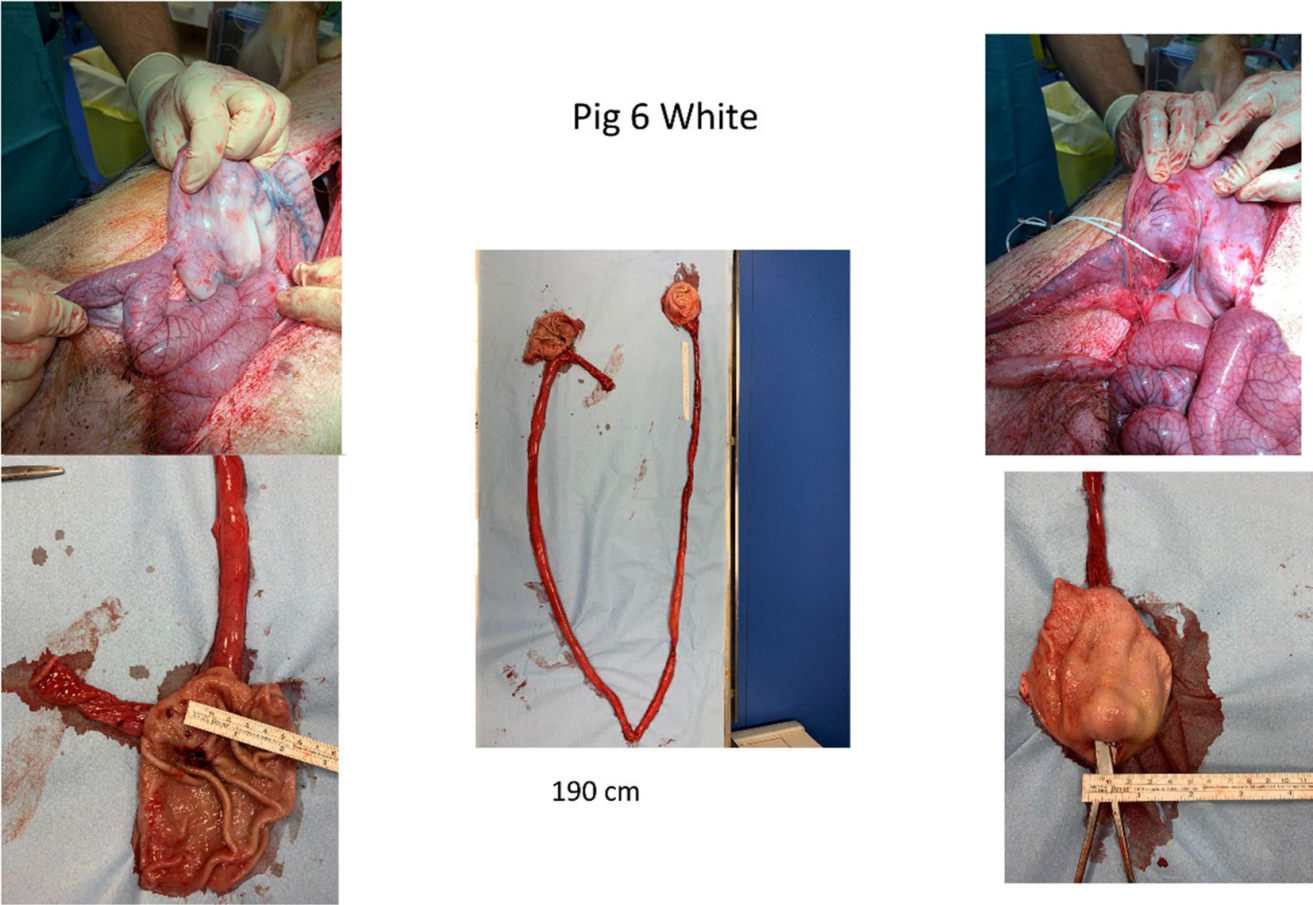
Pylorus, GJA and jejunal limb explanted upon necropsy at 38 weeks.

Length of the bypass from the pylorus to the GJA ranged from 100 to 240 cm (mean 172.5 cm ± 58.5 cm) in four pigs for which the beacon was visualized during GJ-LAMS placement. For one pig (pig #2), the beacon was not visualized during GJ-LAMS placement, so a random intestinal loop had to be grasped during the procedure. The bypass length in this pig was 760 cm. A representative illustration (pig #6) of the explanted pylorus, GJA and jejunal limb is provided in Fig. 5. Another example (pig #3) of pig appearance, endoscopic examination and necropsy findings are documented in Video 5.

### Weight change from baseline to 38 weeks

Mean weight was 26.1 ± 2.7 kg for seven pigs (including one replacement for pig #4) aged 2.5 months at baseline (Table 1). At 38 weeks, the mean weight was 45.6 ± 12.3 kg, representing a mean weight change from baseline of 19.1 ± 11.3 kg (range + 6.3 kg to + 31.7 kg) among five surviving animals. (Two of the seven original pigs died during follow-up and could not be included in the final weight calculations. See “Adverse events”).

**Figure 6:**
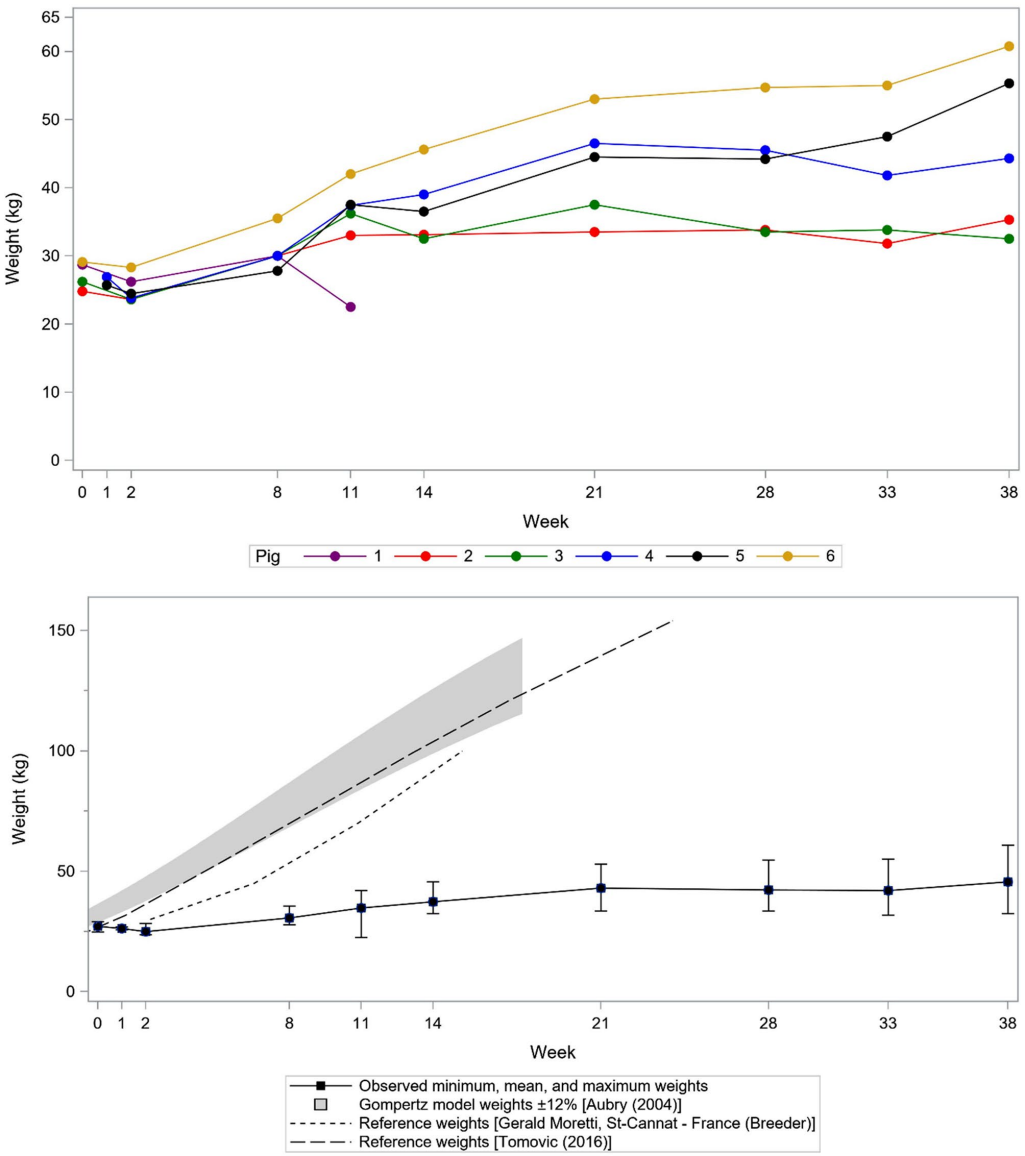
Domestic pig growth curves (**A**) for study animals and (**B**) compared to normal references^14,15^.

Different weight trajectories were observed among the animals (Fig. 6A). Mean weight at Week 2 was significantly smaller compared to Week 0 in all 5 surviving pigs (p = 0.016). Mean weights at Week 8, Week 11, Week 14, Week 21, Week 28, Week 33, and Week 38 were significantly higher than at Week 0 (p = 0.0002–0.019 for individual weeks compared to Week 0), reflecting weight gain by 38 weeks despite weight loss between Weeks 0–2 in all pigs and between Weeks 11–14 in 2 of the 5 surviving pigs. Mean weight of the 5 surviving pigs plateaued after 21 weeks (43.0 kg at 21 weeks, 42.3 kg at 28 weeks, 42.0 kg at 33 weeks, 45.6 kg at 38 weeks).

The trajectory of the mean weight of the five pigs that survived from Baseline to Week 38 (pigs #2–#6) was compared to the normal growth curves obtained for Landrace/Large-White pigs from the breeder (Gerald Moretti, St-Cannat, France), from Aubry et al.^14^ and from Tomovic et al.^15^ (Fig. 6B). Weight gain was significantly lower in the pigs that underwent endoscopic bypass procedures using a GJ-LAMS and DED compared to expected weight for age.

### Adverse events (AEs)

#### One early death

One pig (original pig #4) died on Day 6 from causes not attributed to the devices, but possibly precipitated by operating stress. This animal was underweight (21 kg) and unhealthy at baseline. Air was used instead of CO_2_ for insufflation during the GJ placement procedure, which lasted 1 h and 34 min as the beacon was difficult to find. Upon necropsy, the GJ-LAMS was in place with normal-appearing adjacent tissue. Because no follow-up data were available for this pig, it was not included in the analysis. This pig was replaced with a new healthy pig (animal #4 in Supplementary Table 1) that underwent GJ-LAMS placement on Day 7 and completed the remainder of the study without incident.

#### Other AEs

Three pigs (50%) had diarrhea reported at 3 weeks and 2 (33.3%) had partial duodenal stenosis reported at 11 weeks without associated symptoms (Table 1).

Histopathological examination revealed that 2 of 5 pigs that survived to 38 weeks each had a small (4 mm diameter in pig #2, 5 mm × 2.5 mm diameter in pig #5) abscess in the muscularis externa (“Supplementary Appendix”) near the GJA. These 2 animals showed no clinical signs of infection while alive and no evidence of infection elsewhere on necropsy.

On Day 94 (4 days prior to the planned Week 14 endoscopic evaluation), pig #1 was found dead. At Week 8, this pig was noted to have been eating less for the previous 3 days, and on endoscopic examination was found to have liquid and food gastric stasis, an intact GJ-LAMS for which patency was not evaluable due to food stasis, and a migrated DED (not visible). Pig #1 continued to eat less and had a 7.5 kg (26.1% of baseline weight) weight loss between Week 8 and Week 11. At Week 11, the DED was found to have migrated and become impacted in the GJ-LAMS which was occluded. Both the GJ-LAMS and DED were replaced at Week 11. At the time of death, posthumous endoscopic examination revealed that the new GJ-LAMS and DED were in place with normal-appearing adjacent tissue and no ischemia of the downstream jejunum, so death attributable to previous device failures was not certain.

## Discussion

We conducted the first survival porcine study to test procedural feasibility of a fully endoscopic procedure as a potential alternative to weight-loss surgery. The procedure included establishing controlled length jejunal bypass length, using novel dedicated devices to create a gastrojejunostomy and exclude the duodenum, then removing the devices by 38 weeks. Animal weights were monitored, but weight loss was not a primary goal in this early stage of procedural testing conducted in a growing pig model. Our study advances the field after a 2021 cross-sectional study of 3 pigs used a NOTES-based variation of a gastric bypass procedure^12^. The latter study included creation of a GJA with a LAMS as in our study; however, use of gastroscope-colonoscope localization of the GJA and pyloric closure with an overstitch system in the other study decreases feasibility and safety compared to our methods. Most important, our study is the first to gather essential prospective data on reliability, durability and safety of the devices before they undergo further testing.

Since their development began 70 years ago (1950s)^16^, bariatric operations have evolved to be highly feasible, effective and safe. A 2018 retrospective observational cohort study estimated 30-day rates of major adverse events of 5.0% for RYGB, 2.6% for sleeve gastrectomy and 2.9% for adjustable gastric banding^17^. However, procedural alternatives are needed for patients who are not bariatric surgery candidates, who need a bridge to surgery, or for whom surgical options are not available; for these patients, bariatric endoscopy warrants further study and development as an addition to existing safe and effective bariatric treatments. Especially in patients with morbid obesity (BMI ≥ 40)^18–20^ and in those with comorbidities such as obstructive sleep apnea^21^, perioperative and anesthesia risks are higher for any procedure (surgical or endoscopic). We support continued research on and development of bariatric treatments tailored to patient characteristics and available expertise to maximize long-term efficacy and safety.

Endoscopic treatments are shorter and less invasive than bariatric surgery and avoid the risk of postsurgical ventral hernia occurring secondary to increased intraabdominal pressure in obese patients^22^ However, these procedures have shown varying benefits after extended follow-up. Endoscopically placed 6-month intragastric balloon devices^23,24^, the duodenal-jejunal bypass liner (DJBL)^25,26^, endoscopic sleeve gastroplasty (ESG)^23,27–29^ and aspiration therapy^28^ can achieve short-term weight loss in patients with BMI ≥ 30 kg/m^2^ with or without comorbidities, but weight loss may be diminished or weight may be regained after long-term follow-up^24,30^. Because our animal model was growing domestic pigs, weight gain was expected. We did not compare to control animals in the same study, so it is unknown whether weight attenuation occurred.

The devices we used in the procedure included an indwelling GJ-LAMS and DED. GJ-LAMS migration was observed in three animals (1–2 times each) and DED migration was observed in all animals (1–3 times each in 3 animals). Given that the GJ stenosis was observed by 3 weeks after the Week 14 endoscopic removal of the GJ-LAMS in 2 pigs, GJ-LAMS indwell may need to be longer than 14 weeks. Prior revisions to the uncoated (allows tissue ingrowth into stent mesh) vs. coated portions of the DED and use of mucosal abrasion of the gastric side of the pylorus immediately preceding DED deployment decreased but did not eliminate the rate of DED migration. Substantial weight loss over a shorter period in an adult porcine model would allow removal of the devices earlier to minimize the rate of device-related adverse events including migrations and small bowel wall abscesses observed in this pilot study. While the abscesses were reported as device-related adverse events for optimum safety, these lesions were clinically insignificant because the three affected animals showed no other evidence of infection. Clinically insignificant microabscesses and clinically significant abscesses have been documented for other NOTES-type procedures^31–33^. This finding will be addressed by addition of a microbiological culture protocol in our future studies.

Among the necropsy findings, GJA stricture was of special interest because of its clinical relevance to human gastric bypass surgery. Stenosis of the GJA occurs in approximately 3–27% after gastric bypass, usually within 3 months after surgery, and must be suspected when the patient experiences dysphagia (initially with solids and subsequently with liquids), nausea and vomiting^34^. GJA stenosis/stricture is clinically defined by “a resistance or inability to pass a standard gastroscope through the GJA, suggesting a luminal size of < 10 mm”^34^ and has been classified into grades ranging from mild (10.5 mm endoscope passes through) to complete/near-complete obstruction (nontraversable)^35^ In our study, mean GJA stoma diameter at necropsy was 13.2 ± 2.2 mm (range 10.0–15.0 mm) after 3 of the 5 pigs had GJA dilation at the penultimate study visit (Week 33). This suggests that adequate GJA stoma diameter can be achieved. We plan to perform further studies including GJ-LAMS indwell for at least 6 months, then endoscopic examination at 9 months and one year.

Our study has several strengths and limitations. We demonstrated the technical feasibility of two new indwelling devices specifically designed for use in an endoscopic bariatric or metabolic procedure based on findings from multiple animal laboratories that informed device revisions. Successful GJA creation and duodenal exclusion were achieved. Compared to surgical RYGB or sleeve gastrectomy or to other endoscopic procedures including sleeve gastroplasty procedures, our approach does not include gastric volume reduction; however pyloric closure was included with the intent of augmenting weight loss if migration can be minimized in later procedural development. Our procedure provides a jejunal pull-up bypass anatomy for which a pilot study in adult animals is needed. Two of seven animals died during study follow-up, and necropsy results did not confirm a cause(s) of death. We modified future study protocols to include cultures and a complete blood count after any premature death. Two of five surviving animals were found to have small bowel wall abscesses on histopathology. While these pigs did not show clinical signs of infection, microbiological cultures were not available to confirm whether infection might have occurred in any of the animals in the study. Animal consumption of fortified feed was not quantified, so the role of decreased food intake during the study cannot be estimated. There were no control pigs in this study which was focused primarily on demonstrating technical feasibility and basic safety of a new fully endoscopic procedure. Because the endoscopists were procedural experts with a high level of expertise in NOTES procedures, their results may not be generalizable to endoscopists less experienced in such procedures. Study protocol modifications were done at an early stage of the study because of a systematic migration of the DED which was asymptomatic. This might have impaired the results of the long term follow-up (9 months) by making duodenal exclusion inefficient. Therefore, we decided at the time of the planned early checking to reimplant the DED with mucosal abrasion. The study was funded and sponsored by Boston Scientific Corporation, which built 3 of the 4 novel devices used in this study. The fourth device was built by engineers of the Brussels Medical Device Center, funded by the sponsor. In addition, several sponsor employees contributed to study design, interpretation of study findings, and manuscript writing.

In conclusion, we designed new devices and new stepwise procedure to conduct a pilot study of the first fully endoscopic procedure including a controlled bypass length gastrojejunostomy and duodenal exclusion, achieved in a growing porcine survival study model. This minimally invasive procedure may have future clinical potential for patients with obesity and its metabolic complications; however, it is currently challenged by significant technical and clinical limitations and a high rate of associated adverse events. The results from this study will inform subsequent studies to refine and confirm feasibility of the fully endoscopic procedure and compare the intervention group to a control group in an adult porcine models.

## Supplementary Information

Supplementary Information.

Supplementary Video S1.

Supplementary Video S2.

Supplementary Video S3.

Supplementary Video S4.

Supplementary Video S5.

## Data Availability

The data, analytic methods, and study materials for this study may be made available to other researchers in accordance with the Boston Scientific Data Sharing Policy (http://www.bostonscientific.com/en-US/data-sharing-requests.html).
