# Supplementary material for: First fully endoscopic metabolic procedure with NOTES gastrojejunostomy, controlled bypass length and duodenal exclusion: a 9-month porcine study
Source: Sci Rep. 2022 Jan 7;12:21. doi: 10.1038/s41598-021-02921-9 (PMC8741923; doi:10.1038/s41598-021-02921-9)
Supplement: Supplementary file 1 — Supplementary Information. [file 41598_2021_2921_MOESM1_ESM.docx]

**Supplementary Appendix**

Supplement to: Gonzalez J-M, Ouazzani S, Monino L, et al. First fully endoscopic metabolic procedure with NOTES gastrojejunostomy, controlled bypass length and duodenal exclusion: a 9-month porcine study

**SUPPLEMENTARY MATERIALS AND METHODS**

**Device development and final device designs**

During animal laboratories between April 2017 and September 2019, 4 individual devices (Boston Scientific Corporation, Marlborough, Massachusetts, USA) were developed for a fully endoscopic jejunal bypass with duodenal exclusion. In order of use, these 4 devices are the Enteral Beacon, Atraumatic Grasper, Modified LAMS (GJ-LAMS), and Duodenal Exclusion Device (DED). After design optimization, the 4 devices underwent procedural pilot testing between October 2019 and July 2020.

***Enteral beacon***

The Enteral Beacon design aimed to assure easy and safe GJA positioning at a controlled jejunal depth, then clear visualization during flexible peritoneoscopy.

A compromise between pushability and flexibility was needed to accomplish a controlled-length jejunal bypass. We aimed to select a jejunal limb length for the GJA 150 cm downstream from the pylorus, based on published observational data that this Roux limb length optimizes weight loss in humans without causing malnutrition or persistent diarrhea (Lee S, et al. Curr Surg 2006;63:259-63).

During NOTES-based peritoneoscopy, light emitting diodes (LED) transilluminated the jejunal wall target for the GJA. To reduce heat generation and increase detection, a flashing LED was selected (1.0 Hz, 100 ms per pulse controlled by a programmable oscillator IC). White, green and red lights were tested to optimize visualization. Two approaches were tested: LED capsule attached to the end of a catheter backloaded in the scope vs. LED mounted on a catheter delivered through the scope.

The final Enteral Beacon design was established during 4 animal laboratories in which 6 animals were sacrificed after beacon testing. It was a catheterless guidewire that fits through the operating channel of the endoscope and tracks the small bowel, with a stiffness similar to a standard guidewire (**Figure 1A** in main manuscript). The device has visual markers to measure insertion depth from the dental arcade or pylorus, a flexible tip extension for atraumatic deep enteral advancement, and embedded cyano-acrylate encapsulated Luxeon LXZ1 LEDs approximately 15 cm from the distal end that can be seen in all directions, flashing in red, green or both simultaneously. During peritoneoscopy for initial localization, red light was used for its greater transmission through the jejunal wall and in the peritoneum. Green light was used for close-up viewing to accurately localize the bowel loop for GJ-LAMS distal flange deployment.

***Atraumatic grasper***

The goal was to develop a jejunal limb grasper with optimal jaw length and strength to allow secure grasping and pulling of the jejunal loop to the gastric wall while being atraumatic.

Multiple configurations of the through-the-scope (TTS) flexible Atraumatic Gasper design were evaluated during 3 animal laboratory sessions. Revisions were made after initial handle-breakage and shaft-kinking at the biopsy port while the grasper was pushed down the endoscope.

The final TTS flexible Atraumatic Grasper (**Figure 1B** in main manuscript) is compatible with a 2.8-mm working channel and opens to 16.5 mm at the tip of the jaws when fully opened. The jaws are slightly curved and can be independently rotated.

***GJ-LAMS and delivery system***

For the GJ-LAMS, an existing LAMS design was modified to create concave curvature on the flange. We hypothesized that addition of inward curvature on both flanges creates a more secure apposition between the GJ-LAMS and luminal walls. This was tested by measuring the forces required to pull a GJ-LAMS compared to a standard 20-mm x 10-mm LAMS (AXIOS, Boston Scientific Corporation, Marlborough, Massachusetts, USA) through a 20-mm diameter hole in a membrane mounted on uniaxial tensile tester. The aims were to prevent the deployed distal flange from slipping through the hole in the jejunal wall during pull-back, and to reduce stent migration risk.

This modified GJ-LAMS was evaluated in 4 nonsurvival pigs and 9 survival pigs followed for approximately one month, with standard endoscopy used to periodically evaluate the stent position and peri-implant tissue appearance. After the survival period, the pigs were euthanized and the intact stents were excised.

The final design of the modified 10-mm length and 20-mm stent lumen diameter GJ-LAMS with concave flanges is shown in **Figure 1C** in the main manuscript.

***Duodenal Exclusion Device (DED)***

The requirement of the DED was accurate endoscopic placement across the pylorus without DED migration, resulting in durable duodenal exclusion.

An existing delivery system (Agile TTS Esophageal delivery system, Boston Scientific Corporation, Marlborough, Massachusetts, USA) was used to deploy the DED. Modeled after a LAMS design, the saddle between the 2 flanges was twisted, creating a pinpoint-like closure in the middle of the DED.

Partially silicone-coated DED designs evaluated included a symmetric “Tie Fighter” design, and an asymmetric “Wine Glass” design with a duodenal flange longer than the gastric flange. The coating pattern was optimized to promote partial tissue ingrowth while maintaining structural integrity of the DED.

Two nonsurvival pig studies established the DED delivery feasibility, followed by 2 survival studies (6 pigs and 3 pigs respectively). The fully coated DED migrated proximally into the stomach by 1 week in all animals in the first survival study. However in the second survival laboratory, all partially coated “Tie Fighter” and “Wine Glass” DEDs held position for one month.

The final DED (**Figure 1D** in main manuscript) was a partially covered “Wine Glass” shaped device, with 34-mm diameter flange and 34-mm length.

**Procedural pilot testing**

Endoscopic procedural pilot testing occurred between October 2019 and July 2020.

Details of procedural steps are shown in **Video 1**.

***Animal handling, preoperative care and anesthesia***

The growing (age 2.5 months at baseline) Landrace/Large-White domestic pig model was chosen for adequate handling of animals in serial procedures during a 38-week (approximately 9-month) follow-up period. All animals were assigned an individual identifier and were fasted for 24 hours prior to each procedure.

All endoscopic procedures and evaluations were performed under general anesthesia. Anesthesia was induced by intramuscular injection of 120 mg of azaperone (Stresnil) coupled with 70 mg of ketamine, followed by endotracheal intubation. Anesthesia was maintained by continuous intravenous infusion of propofol (2%) at a rate of 100 mg/hour, and fentanyl was given at a dose of 100 μg/hour for analgesia, with pulse oximetry monitoring of heart rate and oxygen saturation. The animals also received 1g of cefotaxime as antimicrobial prophylaxis preoperatively as well as intraoperatively. All pigs received amoxicillin 500 mg twice daily and Tradonal 100 µg twice daily for 3 days immediately after the baseline GJ stent placement. Following recovery from anesthesia, pain was assessed twice daily by observing animal behavior, and progressive oral intake was resumed. Pigs were given 1 kg daily of a fortified feed with 9.55 MJ/kg energy and 16.5% protein content (Bermond Foods Betail) formulated for pigs less than 2 years of age.

***Creation of GJ anastomosis (Day 1)***

The first step was creation of the GJA with a transmural NOTES approach with CO_2_ insufflation to allow targeted capture of the bypass limb length by the means of a light beacon device. The steps were as follows:

1. *Enteral Beacon endoscopic placement*, targeted to 170-180 cm beyond the pylorus (20-30 cm past the targeted 150 cm) to account for partial distance loss during scope exchange, accomplished by advancement of a colonoscope (Olympus, CF-Q 160, Tokyo, Japan) into the jejunum as far as safely possible, followed by deeper Enteral Beacon device advancement to the desired depth, and removal of the colonoscope while maintaining the Enteral Beacon position**.**
2. *Gastrotomy creation***,** after accessing the gastric lumen with a dual-channel therapeutic gastroscope (Olympus GIF-2TH180, Tokyo, Japan), using a hook-knife.
3. *Access to the peritoneal cavity* with the dual-channel endoscope.
4. *Transillumination* through the small bowel wall by turning on the flashing LED near the distal tip of the Enteral Beacon, to select location for placement of the GJA.
5. *Grasping of the jejunal limb* adjacent to the flashing Enteral Beacon, using the TTS flexible Atraumatic Grasper introduced through the 2.8-mm channel of the endoscope. After the jejunal limb is grasped, the beacon can be turned off and pulled back proximally.
6. *Introduction of electrocautery-enhanced GJ-LAMS* into the 3.7-mm endoscope channel and access the small bowel lumen in close proximity to the Atraumatic Grasper and flashing Enteral Beacon.
7. *GJ-LAMS distal flange deployment* inside the jejunal lumen.
8. *Traction of the small bowel loop to the external gastric wall,* secured by 2 points of control, namely the closed Atraumatic Grasper and the distal flange of the GJ-LAMS.
9. *GJ-LAMS proximal flange deployment* within the stomach, completing creation of the GJA.

***Duodenal exclusion by pyloric closure (Week 2 and Week 11)***

At Week 2, to place the DED, a colonoscope (Olympus, CF-Q 160, Tokyo, Japan) (dual-channel scope optional) was introduced into the stomach and advanced through the pylorus into the duodenum. The distal flange of the DED was deployed in the duodenal bulb. The endoscope and partially deployed DED were carefully pulled back through the pyloric sphincter, and the proximal flange of the DED was deployed in the stomach with the flange seated on the gastric side of the pylorus.

For the replacement procedure at Week 11, electrocoagulation around the circumference on the gastric side of the pylorus was performed using a coagulating grasper forceps (Coagrasper, Olympus, Japan) prior to DED placement to increase tissue ingrowth and reduce the risk of DED migration.

**Protocol adjustments during procedural pilot testing**

***Day 1 and Week 1 for 6 pigs***

One pig died on Day 6 from causes not attributed to the devices, but possibly precipitated by procedural stress. This animal was underweight and unhealthy at baseline. Air was used instead of CO_2_ for insufflation during the GJ placement procedure, which lasted one hour and 34 minutes as the bowel was difficult to grasp. Upon necropsy, the GJ-LAMS was in place with normal-appearing adjacent tissue. Because no follow-up data were available for this pig, it was not included in the analysis. This pig was replaced with a new healthy pig (animal #4 in **Supplementary Table 1**) that underwent GJ-LAMS placement on Day 7 and completed the remainder of the study without incident.

Another pig had food in its stomach on Day 1, so it also underwent GJ-LAMS placement one week later. Thus, 4 of 6 original pigs in this study underwent GJ-LAMS placement on Day 1, and 2 of 6 pigs (one replacement and one original) underwent GJ-LAMS placement at Week 1.

**SUPPLEMENTARY RESULTS**

**Technical success**

***Ability to complete GJA and duodenal exclusion***

The GJ-LAMS placement procedures (including beacon time and stent placement time)

at baseline had mean procedural duration of 35.5 ± 9.2 minutes. Mean duration of DED placement procedures was 4.5 ± 1.9 minutes at Week 2, and 5.5 ± 1.2 minutes at Week 11 (including mucosal abrasion immediately prior to DED deployment) (**Table 1, Figure 3D and 3E**).

Per the study plan (**Figure 2**), half of the pigs (pigs #1, #3, and #5) were intended to undergo endoscopic removal of the GJ-LAMS at Week 14 (**Figure 3B**). In pig #1 at Week 11, the DED was found to have migrated and lodged itself inside the GJ-LAMS, which was easily and atraumatically removed and replaced by a second GJ-LAMS. This pig died prematurely on Day 94 (see “Adverse Events” in main paper). In pigs #3 and #5 at Week 14, the GJ-LAMS was easily and atraumatically removed endoscopically using a rat tooth forceps. The GJA appeared somewhat narrowed upon inspection 3 weeks later, and a second GJ-LAMS was place on Week 17 followed by uncomplicated endoscopic removal on Week 28.

In pigs #2, #4 and #6, the GJ-LAMS was planned to remain indwelling until the end of follow-up. On Week 28 the GJ-LAMS was indwelling in pigs #2 and #6; however, the GJ-LAMS in pig #4 had migrated. On Week 33, the GJ-LAMS had migrated in pig #2 and was easily endoscopically removed in pig #6.

***Removal of DED and endoscopic assessment of the pylorus***

Per the study plan (**Figure 2**), all pigs were intended to undergo endoscopic removal of the DED at Week 28. By Week 11, all DED pyloric plugs were observed to have migrated, so a second DED was placed after superficial mucosal abrasion of the gastric side of the pylorus. These second DEDs were accordingly planned for removal on Week 28. In pig #3 the DED was noted to have migrated when endoscopy was performed to remove its GJ-LAMS at Week 17 and was replaced by a third DED on Week 21. By the end of follow-up, all five pigs were DED-free, after confirmed spontaneous uneventful migration in 3 pigs (pig #2 on Week 33, pig #3 and pig #4 on Week 28) or atraumatic endoscopic removal in two pigs (pig #5 on Week 28, pig #6 on Week 33) (**Figure 3F**). This demonstrated that while all DEDs placed without mucosal abrasion had migrated by 9 weeks after placement, 4 of 6 DEDs placed with mucosal abrasion remained in place for greater than 9 weeks.

**Endoscopic, necropsy and histopathological evaluations at end of study**

Animal appearance, endoscopic examination, and necropsy findings are demonstrated for 1 of the 5 surviving pigs (pig #3) in **Video 2**.

Histopathological examination of necropsy samples of the GJ anastomosis confirmed full-thickness small bowel wall fusion in 1 (20%) pig, fusion of the mucosal and muscularis mucosae but not the muscularis externa in 3 (60%) pigs (**Supplementary Figure 1A and 1C)**, and lack of fusion of all 3 wall layers in 1 (20%) pig.

Two pigs had small (4 mm diameter in pig #2 and 5 mm x 2.5 mm diameter in pig #5) small bowel wall abscesses in the area of the GJ anastomosis (**Supplementary Figure 2 A-C**); both of these pigs had fusion of the mucosal and muscularis mucosae layers but no fusion of the muscularis externa. Mean distance between the 2 muscularis layers was 7.4 (range 0-16) mm, and mean fibrotic thickness was approximately 4.8 (range <1-12) mm. Peritoneal fibrosis was evident in 1 (20%) pig. Otherwise, the peritoneum, pylorus and afferent limb were reported to have normal histopathological appearance (**Supplementary Figure 3**).

**Supplementary Table 1. Pig baseline presentation and procedural results**

| **Animal** | **Distance light beacon past the pylorus (cm)** | **Time for beacon placement (min.)** | **Completion time for GJ anastomosis (min.)** | **Completion time for pyloric closure at wk 11 (min.)** | **GJ-LAMS removed at wk 14** | **All procedures completed** | **Stent-related AEs** | **Comments** |
| --- | --- | --- | --- | --- | --- | --- | --- | --- |
| **1*** | 150 | 19 | 16 | 5 | No | Yes | None | Died on day 94 with no evidence of device failure on postmortem endoscopic examination |
| **2** | unknown | 29 | 8 | 8 | No | Yes | None | Esophageal narrowing led to difficulty advancing light beacon. GJ-LAMS migrated by Week 8, replaced with forceps. |
| **3** | 180 | 12 | 17 | 5 | Yes | Yes | None | Proximal GJ flange did not deploy, stent went into peritoneum, new GJ stent placed |
| **4**^†^ | 185 | 7 | 10 | 5 | No | Yes | None |  |
| **5**^†^ | 150 | 8 | 7 | 5 | Yes | Yes | None | GJ placement postponed due to food in stomach on day 1; successful GJ placement on day 7. |
| **6** | 185 | 15 | 15 | 5 | No | Yes | None | GJ-LAMS migrated by Week 21, was replaced |

GJ gastrojejunal AE adverse event

*Pig #1 died on Day 94; endoscopic examination showed normal GJ stent and pyloric closure device without evidence of tissue inflammation or infection.  The cause of death was unknown but did not seem to result from device failure.

^†^Pigs #4 and #5 had their GJ stents placed on Day 7 (Week 1) because of being a replacement pig, and having food in stomach on Day 1, respectively.

**Supplementary Figure 1. Histopathology of the GJ anastomosis demonstrating fusion between (A) gastric mucosa and jejunal mucosa with (B) nearby superficial ulceration in one pig, and (C) fusion of the muscularis mucosae. (Hematoxylin and eosin [H&E], low [A] and high-power [B,C] magnification)**

**
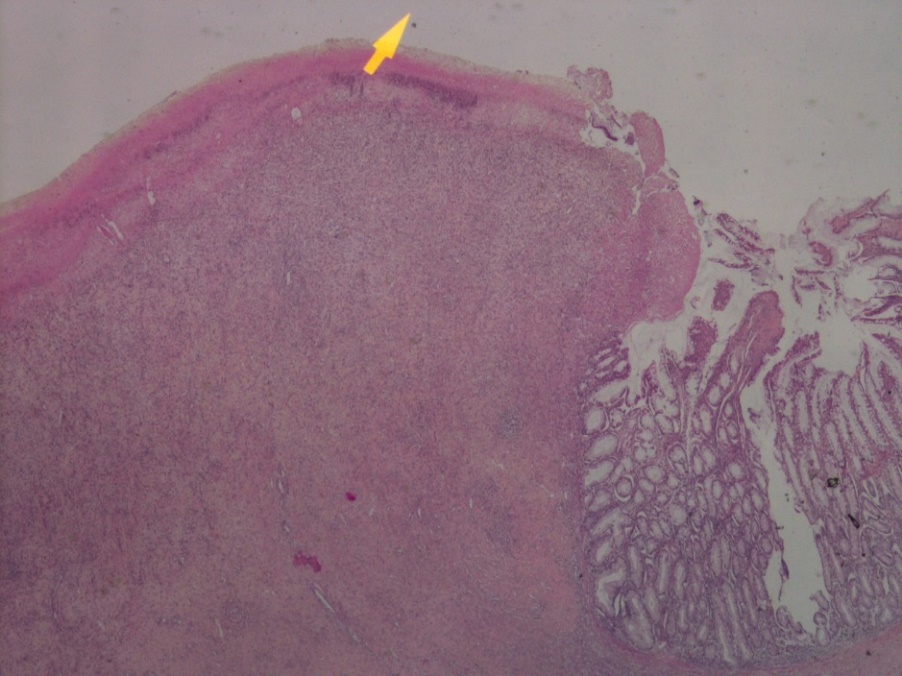

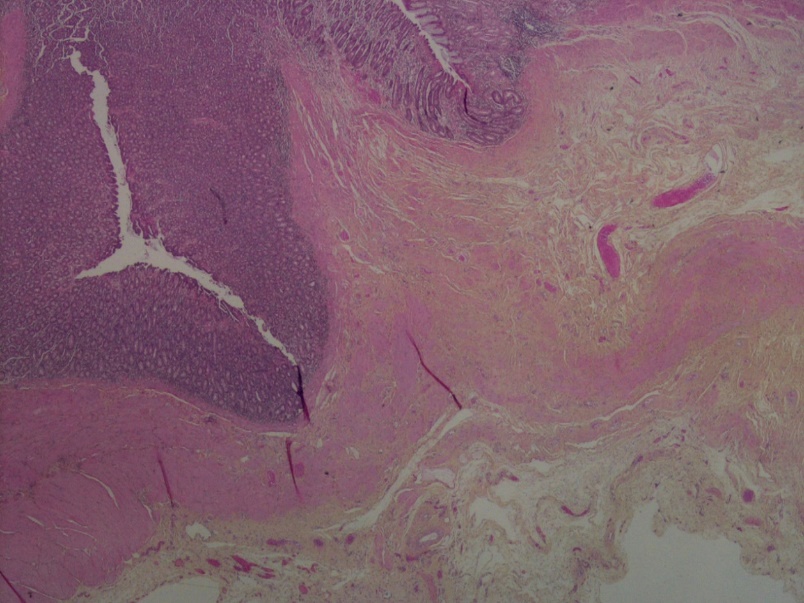

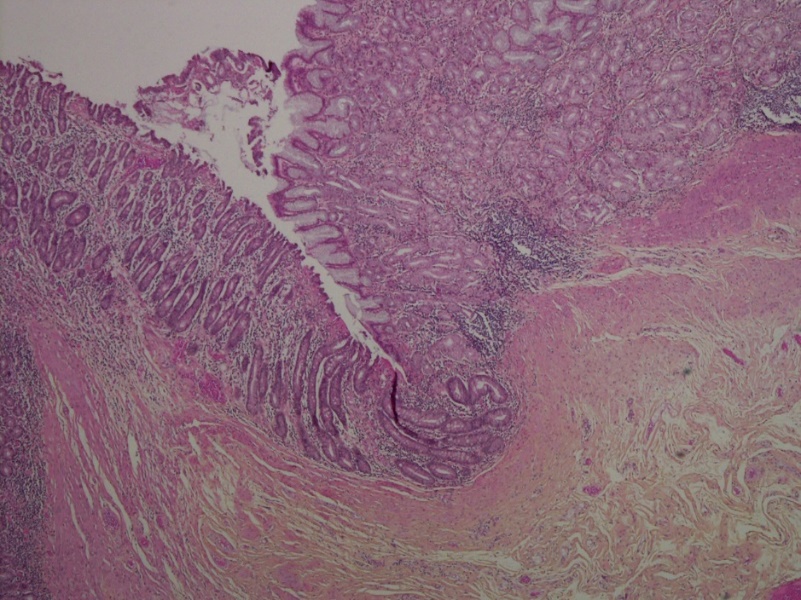
**

C

B

A

**
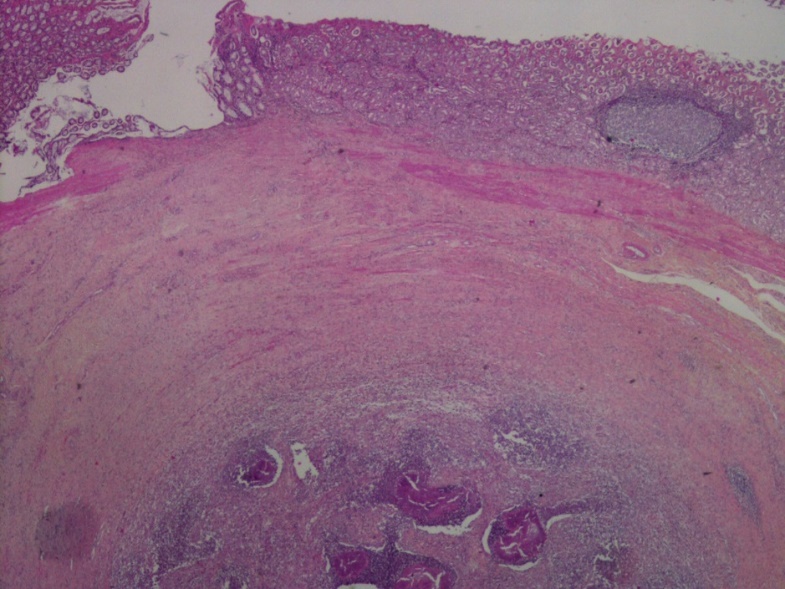

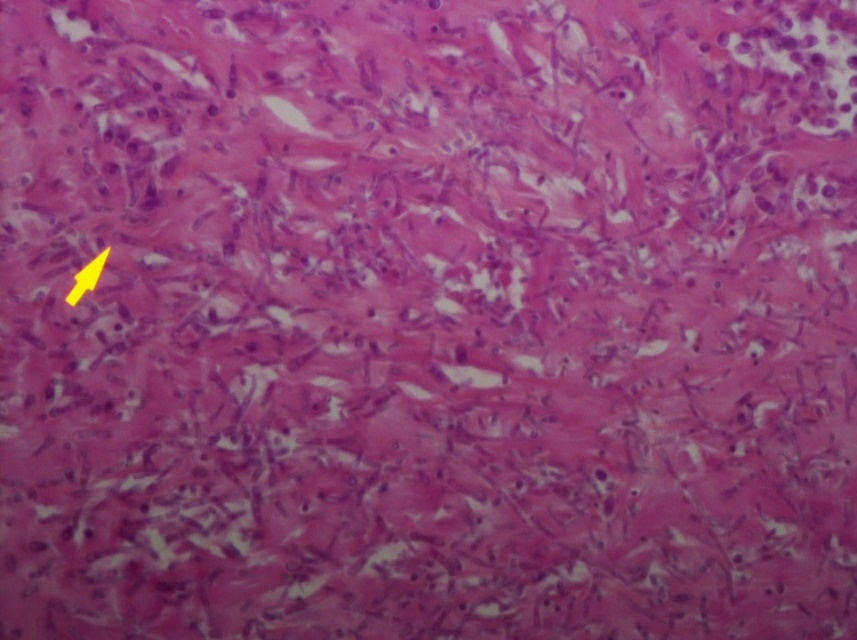

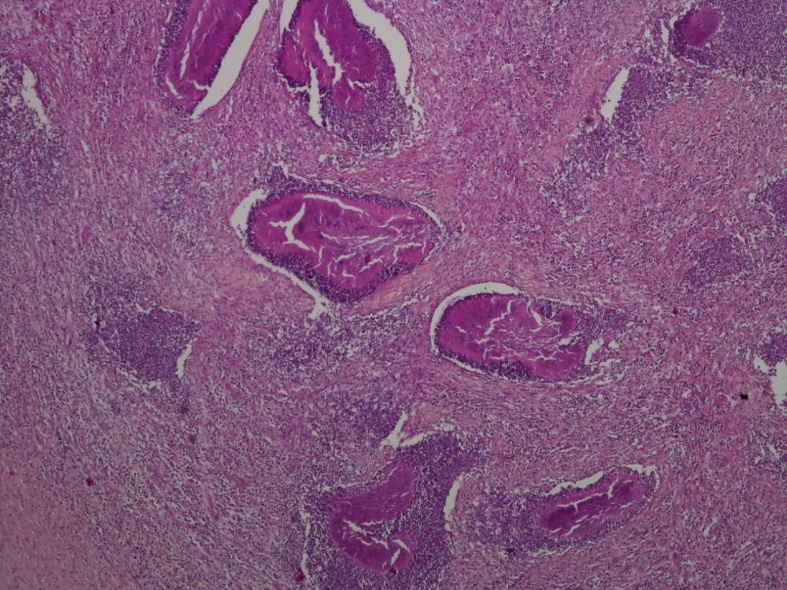
Supplementary Figure 2. Histopathology near the GJ anastomosis demonstrating (A-C) mycotic wall abscesses in the muscularis externa. (Hematoxylin and eosin [H&E], low [A, B] and high-power [C] magnification)**

C

B

A

**
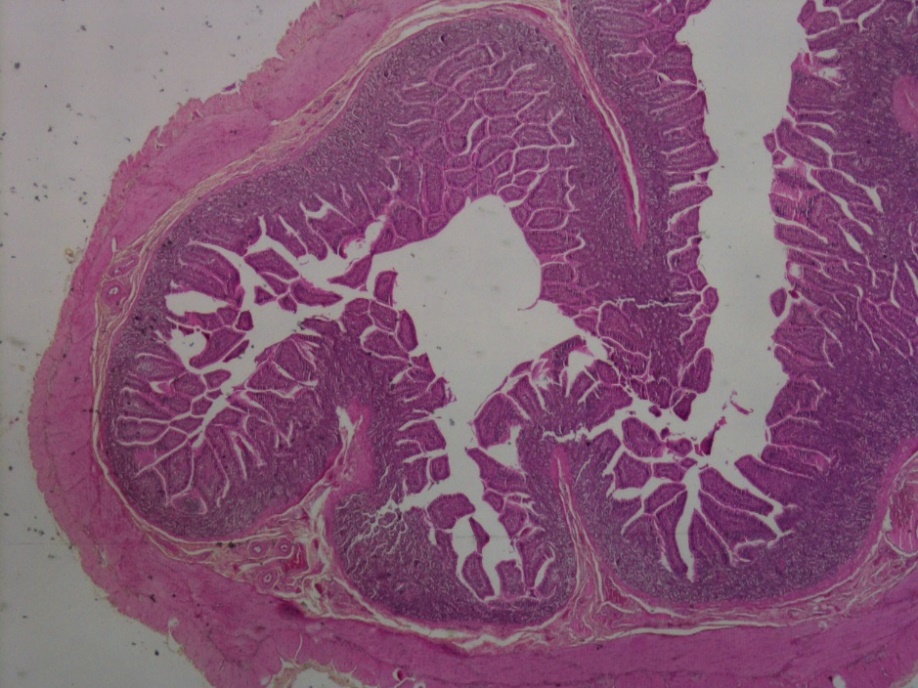

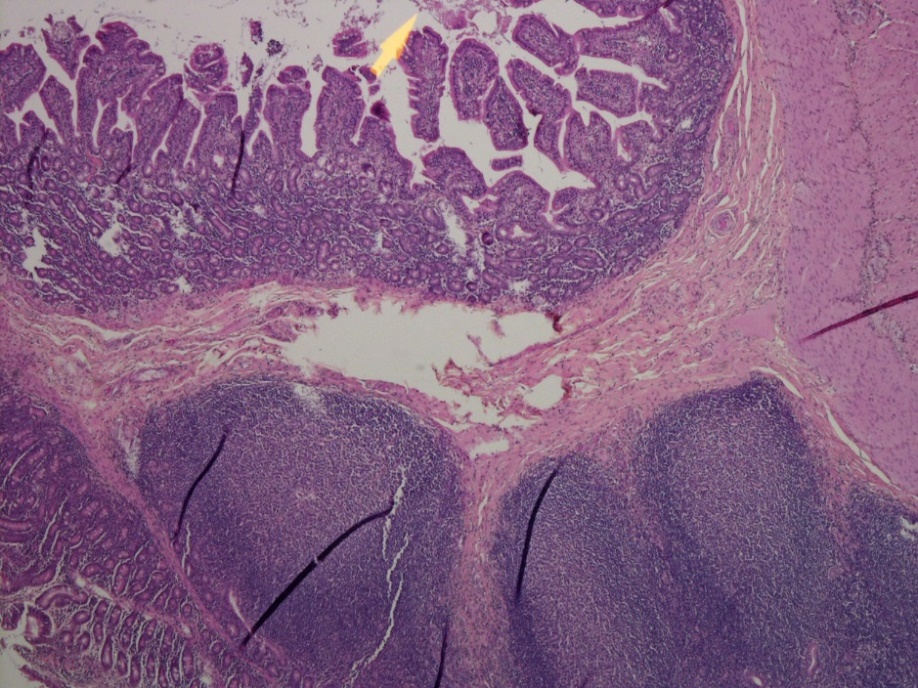
Supplementary Figure 3. Normal histopathology of the (A) afferent and (B) efferent limbs.** **(Hematoxylin and eosin [H&E])**

B

A

**Video 1. Key steps of the natural orifice transluminal endoscopic surgery (NOTES) gastric bypass procedure with controlled limb length.**

**Video 2. Endoscopists are guided by a flashing green enteral light beacon while using a dedicated atraumatic grasper to take hold of a segment of small intestine.**

**Video 3. Deployment of gastrojejunal lumen-apposing metal stent (GJ-LAMS) to form a gastrojejunal anastomosis.**

**Video 4. Deployment of duodenal exclusion device (DED) to close the pylorus.**

**Video 5. Endoscopic examination and gross anatomical findings on necropsy after Pig #3 had a NOTES gastric bypass procedure with controlled limb length.**
